# Functional Evaluation of Cooking‐Mimicking Extracts From Chinese Olive ( *Canarium album* L.) Leaves, Fruits, and Pits Using Cell‐Based and In Silico Analysis

**DOI:** 10.1002/fsn3.70337

**Published:** 2025-05-28

**Authors:** Chun‐Wai Chan, Yu‐Jo Tsai, Ting‐Jang Lu, Yi‐Chun Liao, Shu‐Chen Hsieh

**Affiliations:** ^1^ Institute of Food Science and Technology, College of Bioresources and Agriculture National Taiwan University Taipei Taiwan; ^2^ Department of Biochemical Science and Technology, College of Life Science National Taiwan University Taipei Taiwan

**Keywords:** anti‐cancer, anti‐inflammation, Chinese olive, dietotherapy, phosphofructokinase, phytochemical

## Abstract

Chinese olive (CO; *Canarium album* L.) fruits are rich in phytochemicals with known physiological benefits. However, most studies evaluating the effects of CO have used ethyl acetate, acetone, and chloroform as the extraction solvents, which do not accurately reflect real dietary conditions. Moreover, research on CO leaves and pits remains limited. This study investigated the functional properties of extracts derived from CO leaves, fruits, and pits, prepared under simulated conventional culinary and dietary conditions (including solvents, extract combinations, and heat treatment), referred to as cooking‐mimicking extracts. Our results revealed that most CO extracts reduced the production of nitric oxide in lipopolysaccharide‐stimulated RAW264.7 macrophages and inhibited the proliferation of HCT116 colorectal cancer cells. Combinations of water‐soluble CO extracts exerted a synergistic effect, suppressing cancer cell proliferation. To the best of our knowledge, this study is the first to identify methylellagic acid deoxyhexoside, vitexin 2‐*O*‐rhamnoside, and isovitexin 2‐*O*‐rhamnoside in CO extracts. Molecular docking predicted strong interactions with phosphofructokinase (PFK), a key glycolytic enzyme implicated in cancer cell proliferation. Treatment with CO extracts, including heat‐treated forms, markedly reduced PFK activity and cell viability in HCT116 cells. These findings provide new insights into the bioactive constituents and physiological functions of CO extracts, supporting their potential application in dietotherapy.

## Graphical abstract

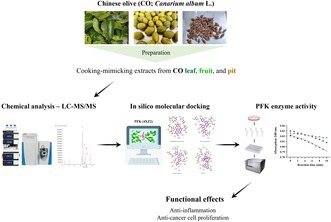

## 1 Introduction

As the number of developed countries and regions continues to rise along with the growing pursuit of a convenient, fast‐paced lifestyle, the consumption of natural foods has markedly declined, resulting in increased dependence on processed foods. A graded and strong association has been noted between the consumption of ultra‐processed food and the incidence of non‐communicable diseases (NCDs), such as cancers, gastrointestinal diseases, obesity, cardiovascular diseases, metabolic disorders, and depression, all of which increase the risk of premature mortality (Monteiro et al. 2019). Studies have reported the effects of microelement insufficiency on the progression of various chronic diseases (Chan et al. 2022; Chan and Lin 2023). Chronic inflammation and oxidative stress are involved in the progression of NCDs, particularly cancer (Khansari et al. 2009). Thus, research is required to determine whether NCDs can be partially prevented and treated using natural plant‐based materials, such as vegetables and fruits, instead of only pharmaceutical interventions, which may lead to varying degrees of adverse side effects.

Studies have demonstrated the physiological benefits of consuming natural polyphenol‐rich foods. Daily intake of one or two fresh kiwifruits for 8 weeks reduced the plasma triglyceride level in healthy individuals, and consumption of 150 g of whole‐berry puree reduced postprandial insulin response to starch‐based foods in healthy women (Brevik et al. 2011; Törrönen et al. 2013). Supplementation with 50 g of freeze‐dried strawberries for 8–12 weeks reduced atherosclerotic risk factors, such as dyslipidemia and circulating adhesion molecules, in individuals with metabolic syndrome and also alleviated pain and inflammation in adults with obesity and knee osteoarthritis (Basu et al. 2010; Schell et al. 2017). In addition, the consumption of 400 mL of pure red grape juice increased antioxidant capacity in healthy individuals (Toaldo et al. 2016).

Chinese olive (CO; *Canarium album* L.), a member of the Burseraceae family, is widely cultivated in Taiwan, southeastern China, and other tropical and subtropical regions of Asia (He and Xia 2007a). Unlike the Mediterranean olive ( *Olea europaea* L.), CO fruits are primarily consumed as food instead of being used for edible oil production (He and Xia 2007a). The fruits, leaves, and pits of CO are all edible. CO fruits are particularly rich in nutrients and are a vital natural source of phytochemicals such as phenolic acids, flavonoids, phenylpropanoids, triterpenoids, and other bioactive compounds (He and Xia 2007a; Yu et al. 2023). Key polyphenols found in CO fruits include gallic acid (GA), ellagic acid, and corilagin, along with flavonoids such as amentoflavone, hyperin, and kaempferol (He and Xia 2007b; He et al. 2008; Yeh et al. 2020). CO is processed into dried powder, seasonings, fruit juice, chewable tablets, and preserves through a well‐established food industry chain (Yu et al. 2023). Dried CO leaves and fruits are commonly used to brew tea. Many individuals in Asia prepare olive soup or wine by using various combinations of CO leaves, fruits, and pits (Kuo et al. 2015; Yu et al. 2023).

In traditional folk medicine, CO fruits are believed to possess therapeutic properties, such as the ability to quench thirst, promote body fluid production, relieve sore throats, mitigate diarrhea, and facilitate detoxification. Yu et al. (2023) summarized the health benefits of different CO fruits components on various diseases. We previously demonstrated that the ethyl acetate fraction of CO fruit extract (CO‐EtOAc) inhibited cancer cell proliferation and tumor growth (Hsieh et al. 2016). Furthermore, we revealed that CO‐EtOAc exhibited anti‐inflammatory, hepatoprotective, and anti‐diabetic effects in diet‐induced obese (DIO) rodent models (Yeh et al. 2017, 2018, 2020).

Other studies have reported that ethyl acetate or chloroform crude extracts of CO fruits exhibit anti‐viral activity (Duan et al. 2013; Yang, Gu, et al. 2018). In addition, the ethyl acetate crude extract exhibited anti‐ *Helicobacter pylori* (Yan et al. 2022), antioxidant, and antiglycation activities (Kuo et al. 2015) and regulated gut microbiota composition in DIO mice (Zhang et al. 2018). Although numerous studies have investigated the physiological benefits of CO fruit components, research on its leaves and pits remains limited. Some studies have examined phenolic phytochemicals in CO leaf extract, revealing antiviral mechanisms against the influenza virus and strong antioxidant activity (Xiao, Cao, et al. 2022; Xiao, Kong, et al. 2022; Zhang and Lin 2008). However, many of these studies used extraction solvents such as methanol, ethyl acetate, and chloroform, which do not accurately reflect conventional culinary practices or real dietary conditions.

Colorectal cancer is among the three most commonly diagnosed cancers worldwide, with an estimated 1.9 million new cases reported in 2022, accounting for 9.6% of all new cancer cases (WHO 2024). A study reported the crucial role of phosphofructokinase (PFK) in promoting the growth and motility of colorectal cancer cells (Lu et al. 2023). Interestingly, our unpublished data suggest the involvement of PFK in CO‐EtOAc‐mediated anticancer properties. Given PFK's central role in glycolysis and its involvement in cancer progression, we conducted an in silico analysis to explore interactions between key compounds in CO extracts and PFK and to investigate their potential inhibitory effects on colorectal cancer cells. In this study, we examined the functional effects of cooking‐mimicking extracts derived from CO leaves, fruits, and pits, prepared under conditions simulating conventional culinary practices and real dietary intake (including solvents, extract combinations, and heat treatment). Through cell‐based functional assays, liquid chromatography–tandem mass spectrometry (LC–MS/MS) analysis, and molecular docking, we elucidated potential mechanisms underlying the anticancer activity of these CO extracts.

## 2 Materials and Methods

### 2.1 General Experimental Instrumentation

A Synergy HT multi‐mode microplate reader equipped with Gen5 software (version 2.09; BioTek Instruments Inc., Winooski, VT, USA) was used to perform nitric oxide (NO), cell viability, PFK activity, and protein content assays. A Luminoskan Ascent microplate luminometer (Thermo Fisher Scientific, Waltham, MA, USA) operated using Ascent software (version 2.6; Ascent, Bristol, UK) was used for luciferase assays.

### 2.2 Chemicals, Solvents, and Reagents

Antibiotics, fetal bovine serum, high‐glucose Dulbecco's modified Eagle's medium (DMEM), and 0.25% trypsin‐ethylenediaminetetraacetic acid were purchased from Thermo Fisher Scientific. Dimethyl sulfoxide (DMSO), lipopolysaccharide (LPS) from *Escherichia coli* O127:B8, methylthiazolyldiphenyl‐tetrazolium bromide (MTT), *N*‐(1‐Naphthyl)ethylenediamine dihydrochloride, phosphoric acid, reserpine, sodium citrate, sodium nitrite, sulfanilamide, *tert*‐butylhydroquinone (*t*‐BHQ), and vitexin 2‐*O*‐rhamnoside were obtained from Sigma‐Aldrich (St. Louis, MO, USA). Corilagin, GA, and theogallin were obtained from ChemScene (Monmouth Junction, NJ, USA). Analytical‐grade organic solvents for extraction and HPLC‐grade solvents for chemical analysis were purchased from J.T. Baker (Phillipsburg, NJ, USA). The extraction buffer was obtained from Abbkine (Atlanta, GA, USA).

### 2.3 Plant Materials and Preparation of CO Leaf, Fruit, and Pit Extracts

**FIGURE 1:**
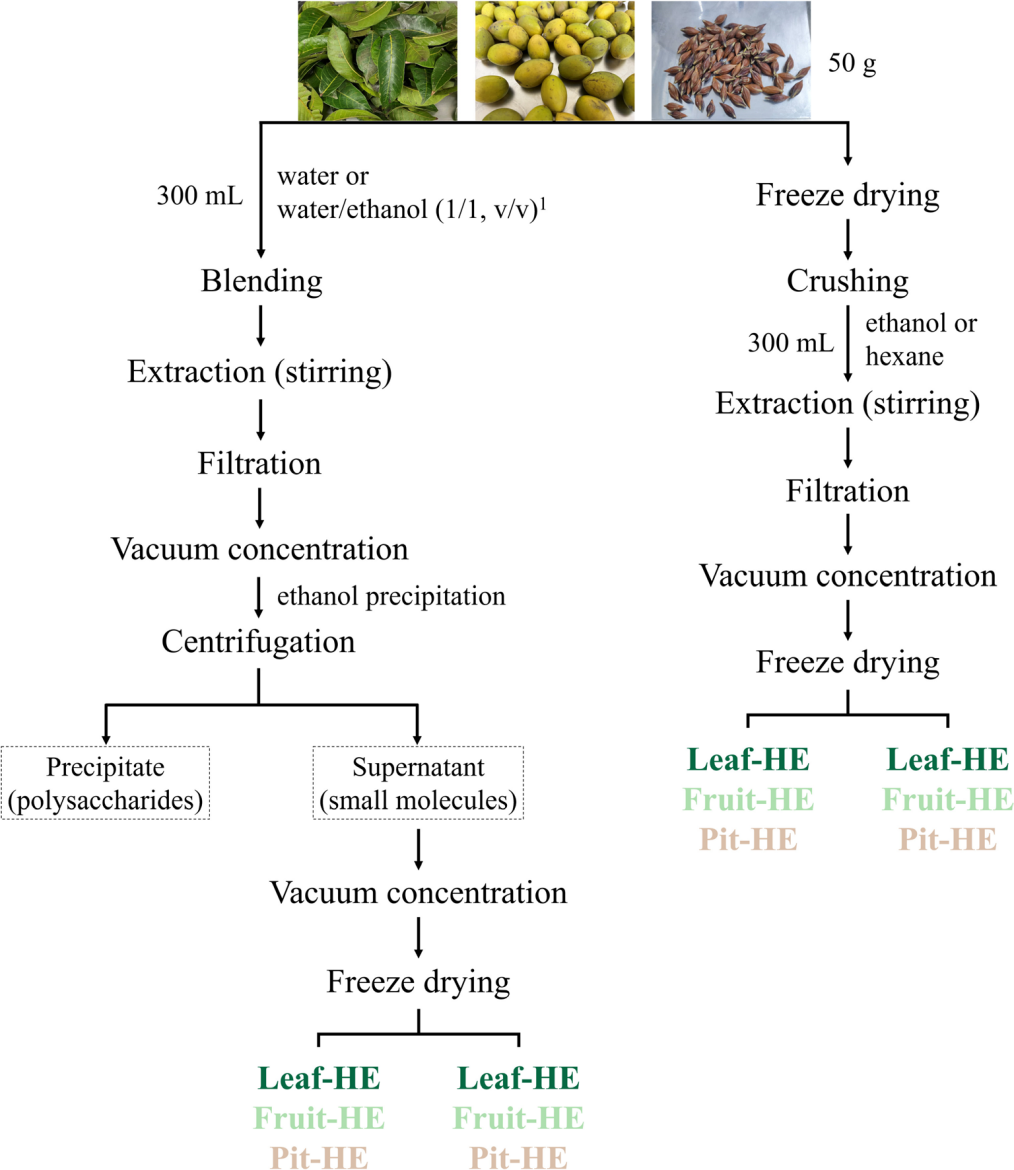
Flowchart illustrating the preparation of CO leaf, fruit, and pit extracts. The water contents of fresh CO leaves, fruit, and pits (48%, 80%, and 34%, respectively) were considered. CO, Chinese olive; EE, ethanol extract; HE, hexane extract; WE, water extract; WEE, water/ethanol (1/1, v/v) extract.

Fresh CO leaves and fruits were purchased from Baoshan Township (Hsinchu County, Taiwan). CO pits were separated from the fruits by using a pitting machine. The preparation of CO leaf, fruit, and pit extracts is illustrated in Figure 1. Fresh samples (50 g) were blended with 300 mL of water or a water/ethanol mixture (1:1, v/v) by using a high‐speed blender, followed by stirring for 1 h. The mixture was then filtered, and the filtrate was concentrated to 100 mL under reduced pressure by using a vacuum evaporator at 60°C. The concentrated solution was mixed with 400 mL of ethanol and left overnight. The ethanol‐treated solution was centrifuged at 2000× *g* for 5 min at 4°C to separate the precipitate (polysaccharides) from the supernatant (small molecules). The supernatant was concentrated and freeze‐dried to obtain CO extracts, including water extracts (WE), namely, Leaf‐WE, Fruit‐WE, and Pit‐WE, as well as water/ethanol (1:1, v/v) extracts (WEE), namely, Leaf‐WEE, Fruit‐WEE, and Pit‐WEE. Additionally, fresh samples (50 g) were freeze‐dried and ground into powder, followed by stirring with 300 mL of ethanol or hexane for 1 h. After filtration, the filtrate was concentrated and freeze‐dried to yield ethanol extracts (EE), namely, Leaf‐EE, Fruit‐EE, and Pit‐EE, as well as hexane extracts (HE), namely, Leaf‐HE, Fruit‐HE, or Pit‐HE. All extraction procedures were performed in the dark at room temperature (25°C). The dry weight and yield percentages of the CO extracts are presented in Table S1. All extracts were stored at −30°C until use.

### 2.4 LC–MS/MS Analysis

Ultra‐high‐performance liquid chromatography (UHPLC) analysis of CO extracts was performed using an UltiMate 3000 UHPLC system (Thermo Fisher Scientific). An ACQUITY UPLC BEH Shield RP18 column (2.1 mm i.d. × 100 mm; 1.7 μm particle size; Waters Corp., Milford, MA, USA) was used. The injection volume was 10 μL, and the column temperature was 40°C. Gradient elution was performed using 0.1% (v/v) formic acid in deionized water (solution A) and 0.1% (v/v) formic acid in acetonitrile (solution B) at a constant flow rate of 0.35 mL/min. The gradient profile was as follows: 98% solution A and 2% solution B at 0 min; 97.5% solution A and 2.5% solution B at 2 min; 97% solution A and 3% solution B at 4 min; 48% solution A and 52% solution B at 34 min; and 10% solution A and 90% solution B from 34.5 to 36.5 min. Absorption spectra were recorded from 190 to 800 nm by using a diode‐array detector. Tandem mass spectrometry (MS) analysis of UHPLC‐separated phenolic flavonoid compounds (PFCs) was performed using a Q‐Exactive mass spectrometer (Thermo Fisher Scientific) equipped with a heated electrospray ionization. Instrument control, data acquisition, and processing were performed using Xcalibur software (version 4.0; Thermo Fisher Scientific). Reserpine was used as the internal standard, and compounds were identified based on retention time and mass‐to‐charge ratio (m/z) compared against reference databases and the literature. Quantification was performed using the standard addition method for GA, theogallin, and vitexin 2‐*O*‐rhamnoside. Relative quantification was performed using vitexin 2‐*O*‐rhamnoside for isovitexin 2‐*O*‐rhamnoside, GA for syringic acid, and corilagin for ellagitannin, ellagic acid deoxyhexoside, ellagic acid pentoside, and methylellagic acid deoxyhexoside.

### 2.5 Cell Cultures

The RAW264.7 murine macrophage cell line (BCRC‐6001) and the HCT116 human colorectal cancer cell line (BCRC‐60349) were obtained from the Bioresource Collection and Research Center (BCRC, Hsinchu, Taiwan). The Ca9‐22 human oral cancer cell line (JCRB0625) was acquired from the Health Science Research Resources Bank (Osaka, Japan). All cell lines were routinely cultured in DMEM supplemented with 10% heat‐inactivated fetal bovine serum and antibiotics (100 units/mL penicillin and 100 μg/mL streptomycin) and incubated at 37°C in a humidified incubator under 5% CO_2_. Cell culture experiments were conducted in three to four independent replicates.

### 2.6 Measurement of NO Concentrations

RAW264.7 cells were seeded in a 96‐well plate at a density of 1.0 × 10^5^ cells/well and incubated with various concentrations of CO extracts (25, 50, 100, and 200 μg/mL) in the presence of the mitogen LPS (100 ng/mL). After 24 h of incubation, NO production was quantified using the Griess assay. Briefly, 50 μL of cell‐free supernatant was mixed with 100 μL of Griess I solution (1% sulfanilamide in 5% phosphoric acid) and 100 μL of Griess II solution (0.1% *N*‐(1‐Naphthyl)ethylenediamine dihydrochloride in 2.5% phosphoric acid). The mixture was incubated in the dark at room temperature for 10 min, and absorbance was measured at 570 nm with a background reference at 690 nm by using a microplate reader. NO concentrations were determined using a sodium nitrite standard curve and normalized to cell viability. Results are expressed as the percentage of NO production relative to the value in the LPS‐treated group.

### 2.7 Luciferase Assay

Our laboratory established a Ca9‐22 cell platform that stably expresses luciferase reporters regulated by nuclear factor erythroid 2‐related factor 2 (Nrf2)‐mediated antioxidant signaling, which is used to screen potential antioxidative food components. Briefly, Ca9‐22 cells were transfected with the plasmid pGL4.18–ARE9–pNL1.3–Nano‐Glo luciferase, which contains an antioxidant response element (ARE) fused to a secretion‐based luciferase reporter gene. The transfected cells are referred to as “ARE‐luciferase” Ca9‐22 cells. These cells were seeded in a 96‐well plate at a density of 1.0 × 10^4^ cells/well and treated with various concentrations of CO extracts (50, 100, and 200 μg/mL) or *t*‐BHQ (10 μM; as a positive control). After 24 h of incubation, corresponding supernatants were collected. Luciferase activity was measured using the Secretory Nano‐Glo Luciferase Assay (Promega, Madison, WI, USA), detected using a microplate luminometer, and normalized to cell viability. Results are expressed as the percentage of luciferase activity relative to the value in the control group.

### 2.8 Cell Viability Assay

HCT116 cells were seeded in a 96‐well plate at a density of 5.0 × 10^3^ cells/well and incubated with various concentrations or combinations of CO extracts for 72 h. Cell viability was assessed using the MTT assay, a colorimetric method for evaluating cell proliferation and survival. Briefly, the culture medium was removed and replaced with serum‐free DMEM containing 0.5 mg/mL MTT. After 2 h of incubation at 37°C, the resulting purple formazan crystals in each well were dissolved in 100 μL of DMSO. Optical density was measured at 570 nm with a background reference at 690 nm by using a microplate reader. Results are expressed as the percentage of cell viability relative to the value in the control group.

### 2.9 In Silico Molecular Docking Analysis

The X‐ray crystal structure of human PFK complexed with adenosine diphosphate (ADP) and fructose 6‐phosphate was obtained from the Research Collaboratory for Structural Bioinformatics Protein Data Bank (ID: 4XZ2; https://www.rcsb.org/structure/4XZ2). The PFK structure was used as the target protein for docking analysis and was processed using the PyMOL platform (Schrödinger Inc., NY, USA) to remove cocrystallized ligands. Structures of small‐molecule ligands present in CO water extracts (Leaf‐WE, Fruit‐WE, and Pit‐WE) were retrieved from the PubChem database. For docking, both the protein and ligand structures were converted to the pdbqt file format by using PyRx with the AutoDock Vina Tools package, following standard docking parameters (Morris et al. 2009). The output docking scores were reported as binding affinities (kcal/mol). Adenosine triphosphate (ATP) was used as a reference ligand.

### 2.10 PFK Activity Assay

HCT116 cells were seeded at a density of 2.0 × 10^6^ cells per 6‐cm dish and treated with DMSO (0.5%; solvent control), sodium citrate (20 mM; as a positive control), or CO water extracts (Leaf‐WE, Fruit‐WE, and Pit‐WE; 200 μg/mL) for 24 h. PFK activity was measured using a commercial kit (Abbkine, Atlanta, GA, USA) on the basis of enzymatic conversion of fructose‐6‐phosphate and ATP into fructose‐1,6‐bisphosphate and ADP, respectively, accompanied by the oxidation of NADH to NAD^+^ through enzyme cascades and lactate dehydrogenase. Because NADH exhibits maximum absorbance at approximately 340 nm, PFK activity was calculated by monitoring the reduction in absorbance at this wavelength over time. Briefly, cells were washed twice with ice‐cold phosphate‐buffered saline, and 500 μL of extraction buffer was added for ultrasonic disruption in an ice bath by using a sonicator (power, 50%; ultrasonication duration, 3 s, cool‐down time, 7 s; repeat, 20 times; Qsonica, Newtown, CT, USA). The lysates were centrifuged at 8000× *g* for 5 min at 4°C to collect the supernatant. Each sample (10 μL) and enzyme mix (20 μL) were added to 170 μL of substrate mix reagent in a 96‐well plate. Absorbance was measured at 340 nm kinetically from baseline (*A*_0_) to 10 min (*A*_10_) by using a microplate reader. The change in absorbance (Δ*A* = *A*_1_ − *A*_10_) was used to calculate PFK activity by using the following formula: PFK activity = 642 × Δ*A* × dilution factor. Protein concentrations in the supernatants were determined using the bicinchoninic acid assay (Thermo Fisher Scientific) to normalize PFK activity. Results are expressed as the percentage of PFK activity relative to the value in the DMSO‐treated group.

### 2.11 Statistical Analysis

Results are expressed as mean ± standard deviation (SD) values. Differences between groups were evaluated using an unpaired two‐tailed Student's *t*‐test. A *p* value of 0.05–0.1 indicated a tendency toward statistical significance, whereas a *p* value of < 0.05 indicated statistical significance. All statistical analyses and graphical representations were performed using Prism (version 8.0.2; GraphPad Software, La Jolla, CA, USA).

## 3 Results

### 3.1 Anti‐Inflammatory Effects of CO Leaf, Fruit, and Pit Extracts on RAW264.7 Macrophages

**FIGURE 2:**
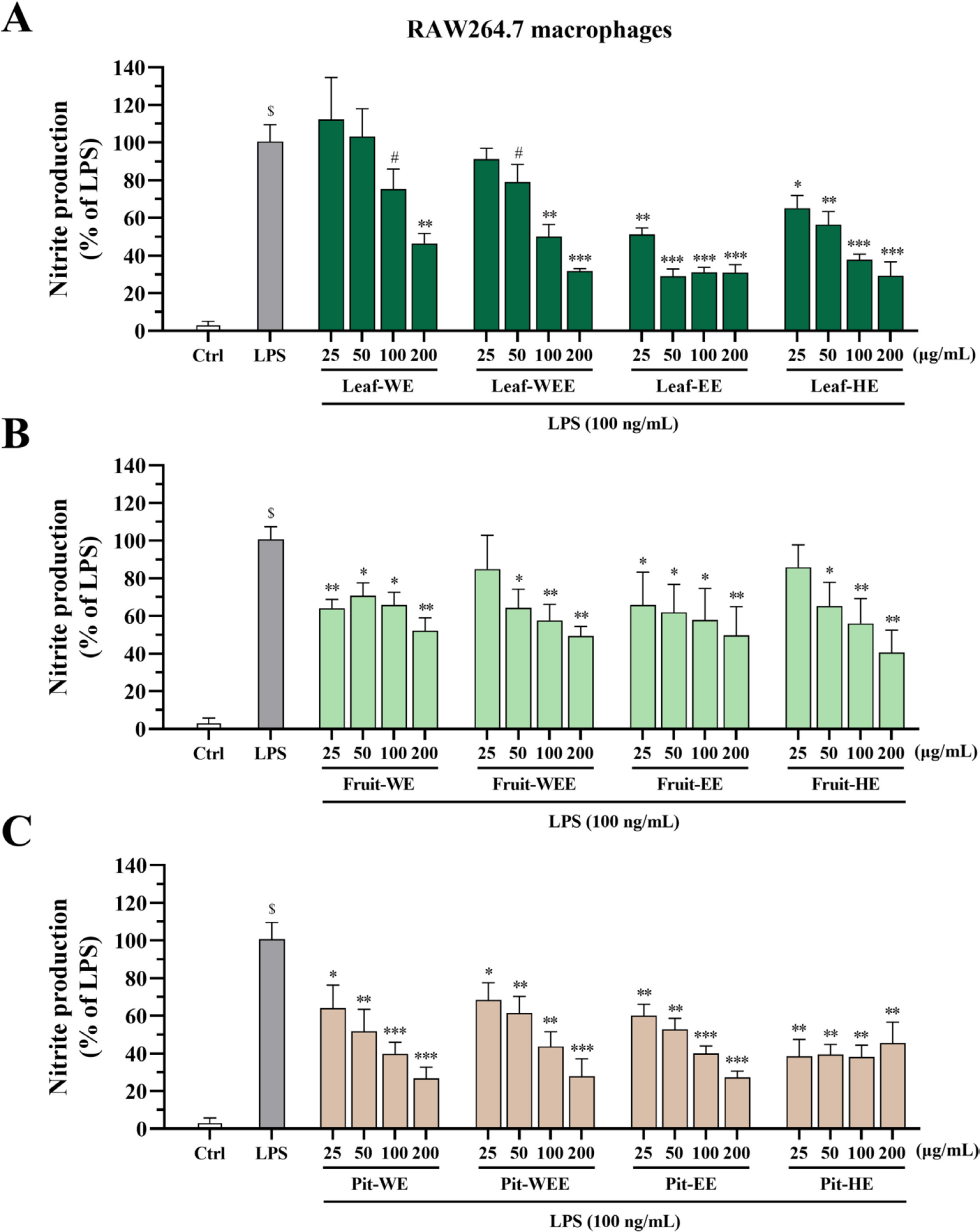
Effects of CO leaf, fruit, and pit extracts on nitric oxide (NO) production in RAW264.7 macrophages. Cells were incubated with dimethyl sulfoxide (DMSO, 0.5%; solvent control) or various concentrations (25, 50, 100, and 200 μg/mL) of CO (A) leaf, (B) fruit, or (C) pit extracts and cotreated with lipopolysaccharide (LPS, 100 ng/mL). After 24 h, NO concentrations in the supernatants were determined using the Griess assay and normalized to cell viability. Data are presented as mean ± standard deviation (SD) values obtained from four independent experiments. ($) *p* < 0.001 versus the control group; (#) 0.05 < *p* < 0.1, (*) *p* < 0.05, (**) *p* < 0.01, and (***) *p* < 0.001 versus the LPS‐treated group; unpaired two‐tailed Student's *t*‐test. CO, Chinese olive; EE, ethanol extract; HE, hexane extract; WE, water extract; WEE, water/ethanol (1/1, v/v) extract.

To examine the anti‐inflammatory effects of CO extracts, we used LPS‐stimulated RAW264.7 cells as a screening platform. As shown in Figure 2, the LPS‐treated groups exhibited significantly higher concentrations of NO, a proinflammatory marker, than did the control groups, confirming effective activation of RAW264.7 cells by 100 ng/mL LPS. Cells were incubated for 24 h with various concentrations of CO leaf extracts (25, 50, 100, and 200 μg/mL), WE, WEE, EE, or HE, in combination with LPS. None of these concentrations exhibited cytotoxicity, as confirmed by cell viability assays (Figure S1). Treatment with Leaf‐WE (200 μg/mL), Leaf‐WEE (100 and 200 μg/mL), Leaf‐EE (25, 50, 100, and 200 μg/mL), and Leaf‐HE (25, 50, 100, and 200 μg/mL) significantly reduced NO production in LPS‐stimulated RAW264.7 cells (Figure 2A; dark green bars). Similarly, Fruit‐WE and Fruit‐EE at all tested concentrations, along with Fruit‐WEE and Fruit‐HE at 50, 100, and 200 μg/mL concentrations, significantly reduced the production of NO (Figure 2B; bright green bars). In addition, Pit‐WE, Pit‐WEE, Pit‐EE, and Pit‐HE significantly inhibited NO production at all tested concentrations (Figure 2C; brown bars). These findings suggested that extracts from CO leaves, fruits, and pits exerted potent anti‐inflammatory effects by suppressing LPS‐induced NO production in RAW264.7 macrophages.

### 3.2 Antioxidant Effects of CO Leaf, Fruit, and Pit Extracts on ARE−Luciferase Ca9‐22 Cells

**FIGURE 3:**
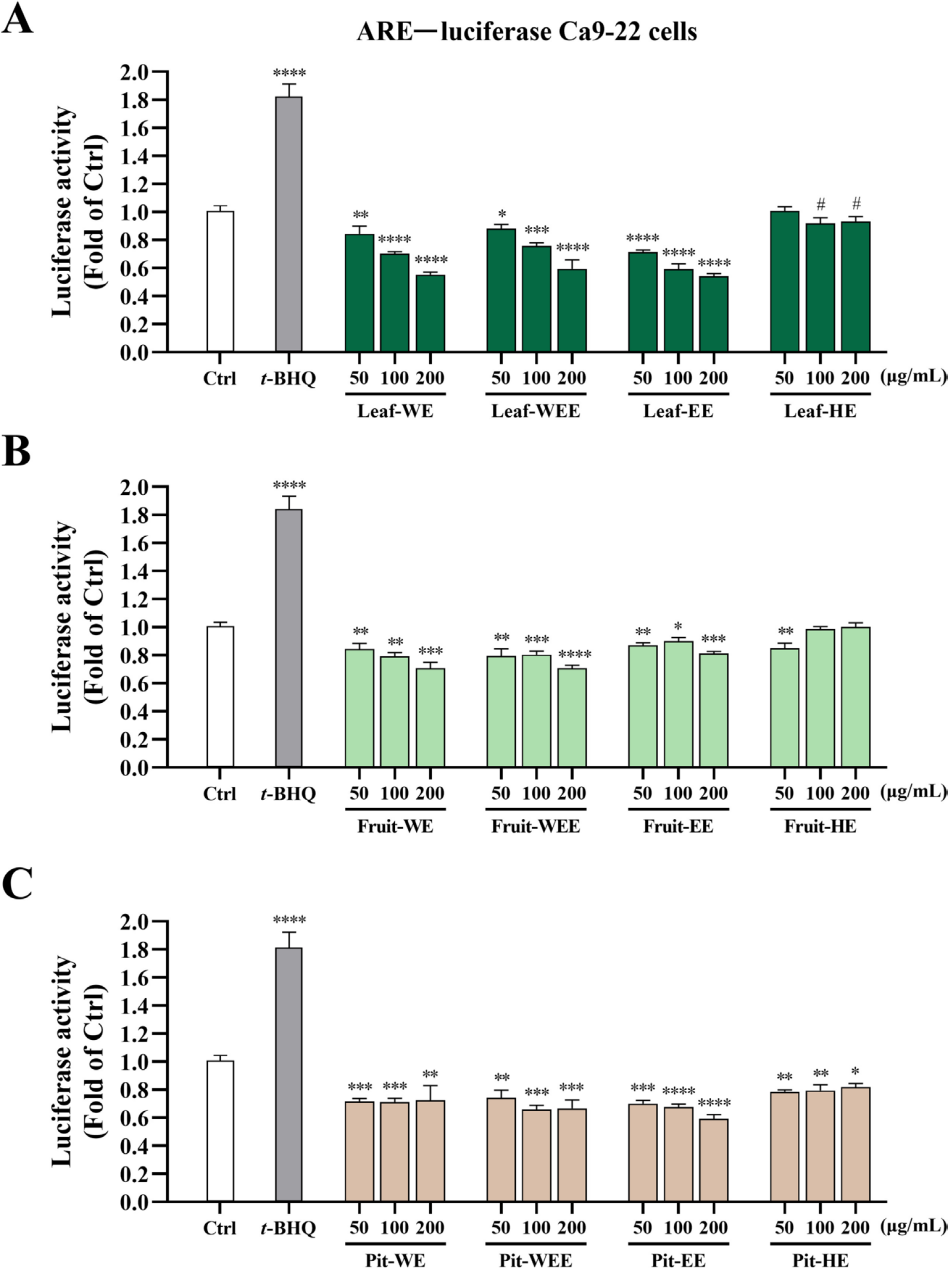
Effects of CO leaf, fruit, and pit extracts on luciferase activity of ARE−luciferase Ca9‐22 cells. Cells were incubated with DMSO (0.5%; solvent control) or *tert*‐butylhydroquinone (*t*‐BHQ; 10 μM; as a positive control), or various concentrations (50, 100, and 200 μg/mL) of CO (A) leaf, (B) fruit, or (C) pit extracts for 24 h. The supernatants were assayed for luciferase activity and normalized to the cell viability. Data are presented as mean ± SD values obtained from four independent experiments. (#) 0.05 < *p* < 0.1 (*) *p* < 0.05, (**) *p* < 0.01, (***) *p* < 0.001, and (****) *p* < 0.0001 versus the control group; unpaired two‐tailed Student's *t*‐test. CO, Chinese olive; EE, ethanol extract; HE, hexane extract; WE, water extract; WEE, water/ethanol (1/1, v/v) extract.

The antioxidant effects of CO extracts were assessed using the ARE‐luciferase Ca9‐22 cell platform. As shown in Figure 3, treatment with *t*‐BHQ (10 μM) significantly increased luciferase activity compared with the control group, confirming the function of *t*‐BHQ as a Nrf2 agonist (Zhao et al. 2020) and validating the suitability of the ARE‐luciferase Ca9‐22 platform for screening potential antioxidant compounds. By contrast, despite the absence of cytotoxicity (cell viability > 90% across all groups, Figure S2), treatment with various concentrations (50, 100, and 200 μg/mL) of CO leaf extracts (Leaf‐WE, Leaf‐WEE, Leaf‐EE, and Leaf‐HE; Figure 3A; dark green bars), CO fruit extracts (Fruit‐WE, Fruit‐WEE, Fruit‐EE, and Fruit‐HE; Figure 3B; bright green bars), and CO pit extracts (Pit‐WE, Pit‐WEE, Pit‐EE, and Pit‐HE; Figure 3C; brown bars) did not increase luciferase activity in ARE‐luciferase Ca9‐22 cells. These results indicated that CO extracts lacked Nrf2‐mediated antioxidant activity under the conditions tested.

### 3.3 Antiproliferative Effects of CO Leaf, Fruit, and Pit Extracts on HCT116 Cells

**FIGURE 4:**
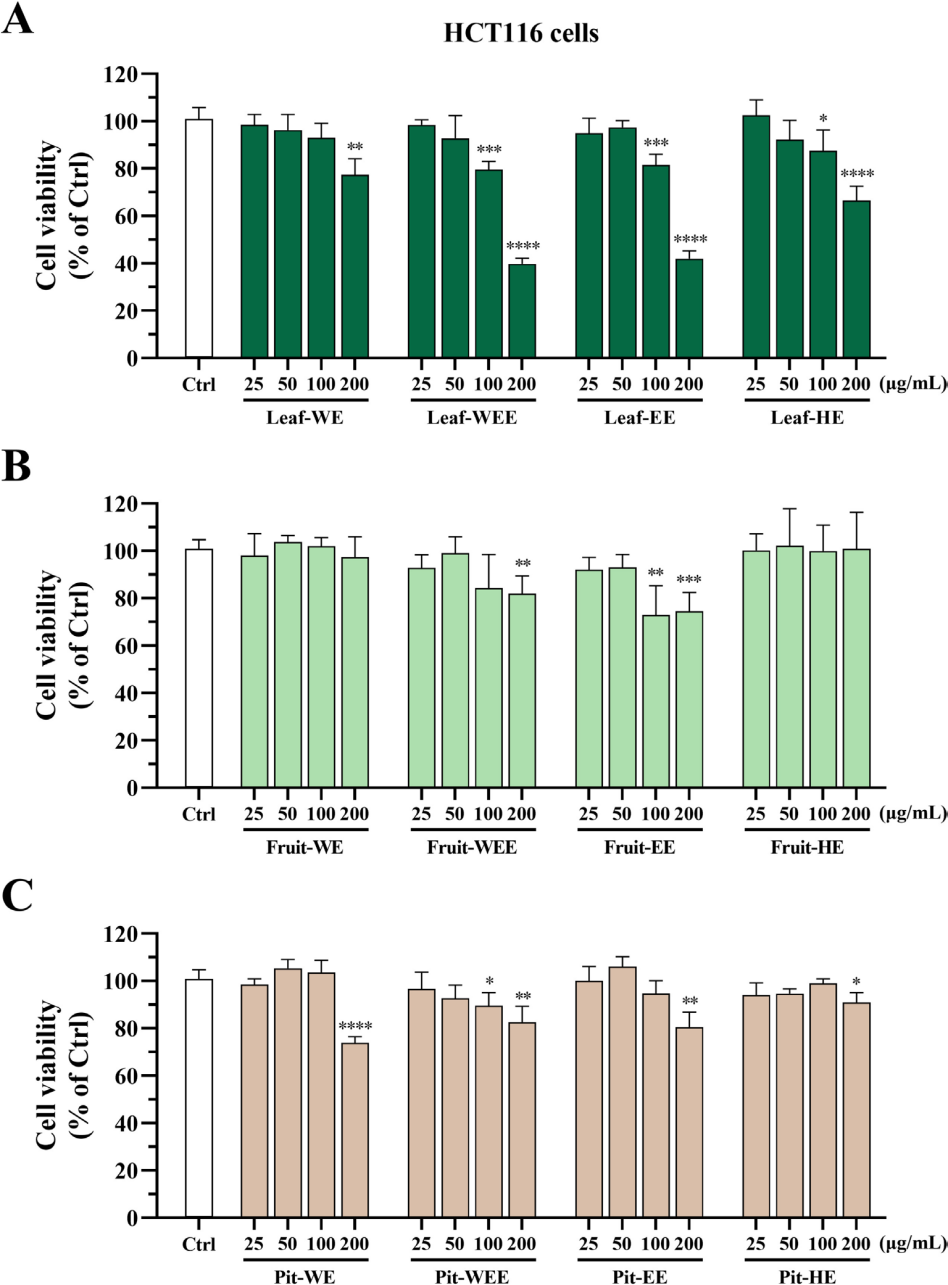
Effects of CO leaf, fruit, and pit extracts on cell viability of HCT116 cells. Cells were incubated with DMSO (0.5%; solvent control) or various concentrations (25, 50, 100, and 200 μg/mL) of CO (A) leaf, (B) fruit, or (C) pit extracts for 72 h. Cell viability was assessed using the MTT assay. Data are presented as mean ± SD values obtained from four independent experiments. (*) *p* < 0.05, (**) *p* < 0.01, (***) *p* < 0.001, and (****) *p* < 0.0001 versus the control group; unpaired two‐tailed Student's *t*‐test. CO, Chinese olive; EE, ethanol extract; HE, hexane extract; WE, water extract; WEE, water/ethanol (1/1, v/v) extract.

To evaluate the antiproliferative effects of CO extracts, HCT116 human colorectal cancer cells were used as a screening platform. Cells were treated with CO leaf, fruit, and pit extracts at concentrations of 25, 50, 100, and 200 μg/mL, and cell viability was assessed using the MTT assay after 72 h of incubation. Treatment with Leaf‐WE (200 μg/mL), Leaf‐WEE, Leaf‐EE, and Leaf‐HE (100 and 200 μg/mL) markedly suppressed cell proliferation compared with the control group (Figure 4A; dark green bars). Fruit‐WEE (200 μg/mL) and Fruit‐EE (100 and 200 μg/mL) also significantly reduced cell viability, whereas Fruit‐WE and Fruit‐HE exerted no observable effect (Figure 4B; bright green bars). The highest concentration (200 μg/mL) of pit extracts (Pit‐WE, Pit‐WEE, Pit‐EE, and Pit‐HE) significantly reduced cell viability (Figure 4C; brown bars). At a concentration of 200 μg/mL, none of the CO extracts exhibited significant cytotoxicity against CCD841 CoN human normal colon epithelial cells (Figure S3). These findings suggested that CO leaf, fruit, and pit extracts exerted specific antiproliferative effects on HCT116 colorectal cancer cells.

### 3.4 Antiproliferative Effects of CO Water Extract Combinations on HCT116 Cells

**FIGURE 5:**
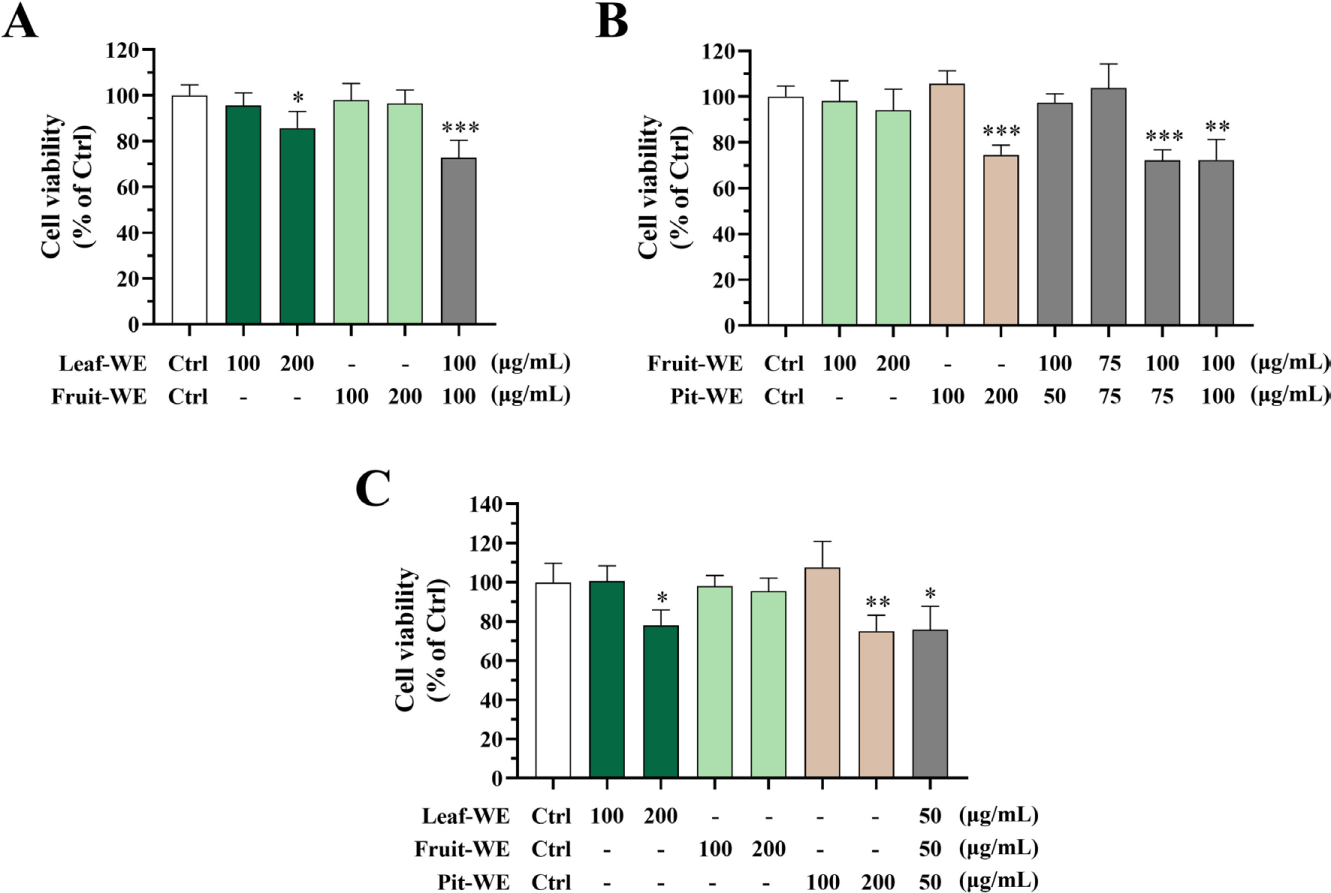
Effects of CO leaf, fruit, and pit water extract combinations on cell viability of HCT116 cells. Cells were incubated with DMSO (0.5%; solvent control) or various concentrations (50–200 μg/mL) of CO (A) Leaf‐WE and Fruit‐WE, (B) Fruit‐WE and Pit‐WE, or (C) Leaf‐WE, Fruit‐WE, and Pit‐WE combinations. After 72 h of incubation, cell viability was assessed using the MTT assay. Data are presented as mean ± SD values obtained from three independent experiments. (*) *p* < 0.05, (**) *p* < 0.01, and (***) *p* < 0.001 versus the control group; unpaired two‐tailed Student's *t‐t*est. CO, Chinese olive; WE, water extract.

To investigate the most common and practical dietary applications of CO, we combined the water extracts of CO leaves, fruits, and pits, simulating conventional culinary and real dietary conditions. HCT116 cells were treated with various concentrations and combinations of CO water extracts for 72 h, and cell viability was evaluated using the MTT assay. As shown in Figure 5A, treatment with Leaf‐WE (100 μg/mL) or Fruit‐WE (100 μg/mL) alone did not affect cancer cell proliferation. However, cotreatment with Leaf‐WE and Fruit‐WE (each at 100 μg/mL), mimicking olive tea, significantly reduced cell viability. Similarly, neither Fruit‐WE (100 and 200 μg/mL) nor Pit‐WE (100 μg/mL) alone exhibited inhibitory effects, whereas the combination of Fruit‐WE (100 μg/mL) and Pit‐WE (75 or 100 μg/mL), mimicking olive soup, significantly reduced cell viability (Figure 5B). In addition, a combination of Leaf‐WE, Fruit‐WE, and Pit‐WE (each at 50 μg/mL), also mimicking olive soup, markedly reduced cancer cell proliferation (Figure 5C). These findings suggested that combinations of CO water extracts exhibited synergistic antiproliferative effects on HCT116 colorectal cancer cells.

### 3.5 Analysis of Phenolic Flavonoid Compounds in CO Water Extracts

**FIGURE 6:**
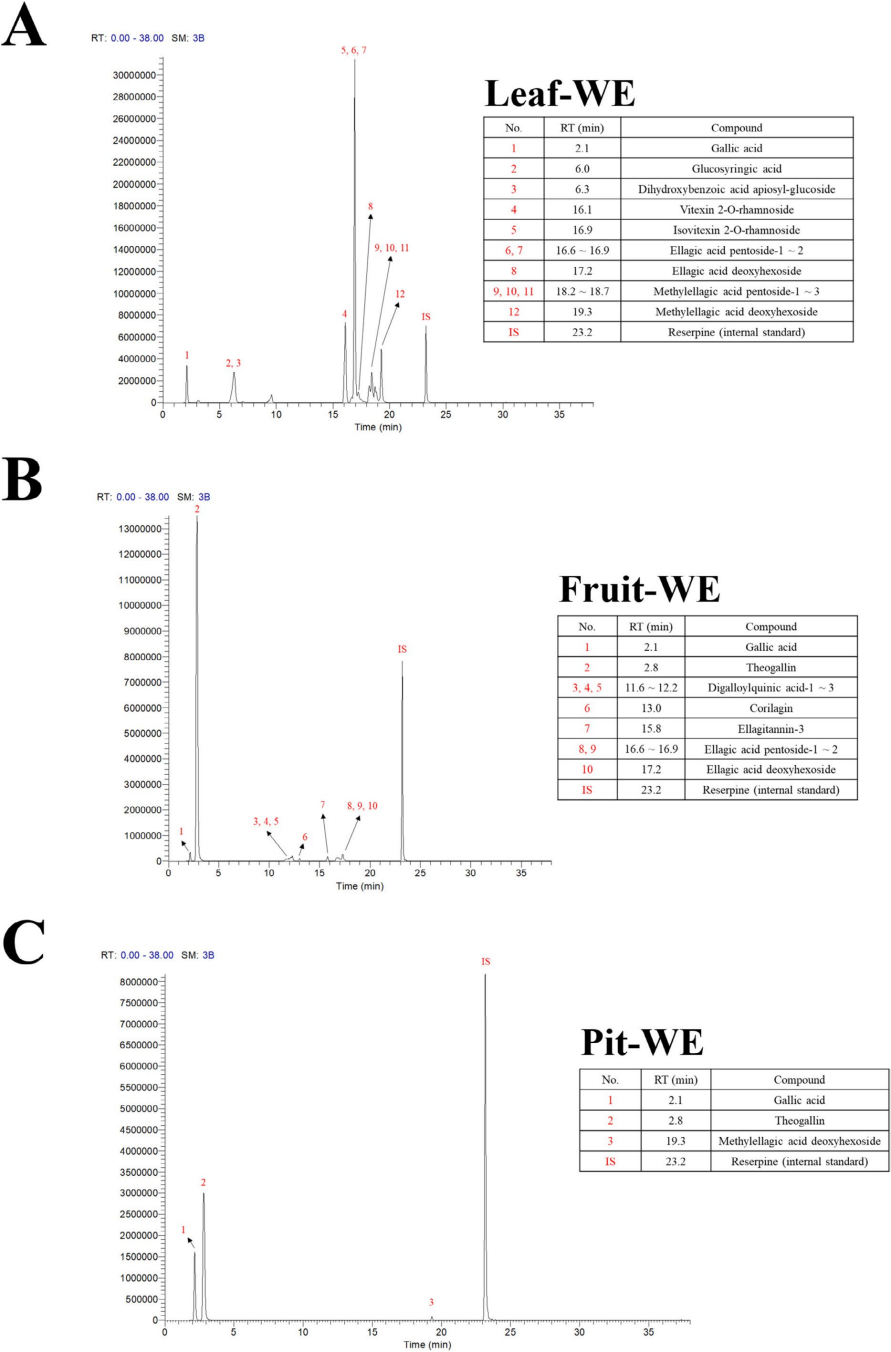
LC–MS/MS pattern of CO water extracts. Extracted ion chromatograms of phenolic flavonoid compounds in CO (A) Leaf‐WE, (B) Fruit‐WE, and (C) Pit‐WE. Reserpine was used as the internal standard. CO, Chinese olive; WE, water extract.

To explore the potential mechanism underlying the synergistic antiproliferative effect of CO water extracts combinations on colorectal cancer cells, we analyzed the small‐molecule compounds, specifically PFCs, in CO extracts through LC–MS/MS. As shown in Figure 6A, Leaf‐WE contained 24.5 mg/g of the total PFCs, with methylellagic acid deoxyhexoside (5.5 mg/g; 22.4%), isovitexin 2‐*O*‐rhamnoside (5.0 mg/g; 20.3%), and vitexin 2‐*O*‐rhamnoside (3.5 mg/g; 14.2%) as the major components. Among the CO water extracts, Fruit‐WE had the highest total PFC content at 515.2 mg/g, primarily composed of theogallin (474.3 mg/g; 92.1%), ellagic acid pentoside (8.7 mg/g; 1.7%), and ellagitannin‐3 (7.9 mg/g; 1.5%; Figure 6B). Pit‐WE contained 48.2 mg/g of total PFCs, including theogallin (41.5 mg/g; 85.9%), GA (4.7 mg/g; 9.7%), and methylellagic acid deoxyhexoside (2.1 mg/g; 4.4%; Figure 6C). These results revealed the PFC composition of CO water extracts and highlighted their potential contribution to the observed inhibition of colorectal cancer cell proliferation.

### 3.6 Results of In Silico Molecular Docking Analysis

**FIGURE 7:**
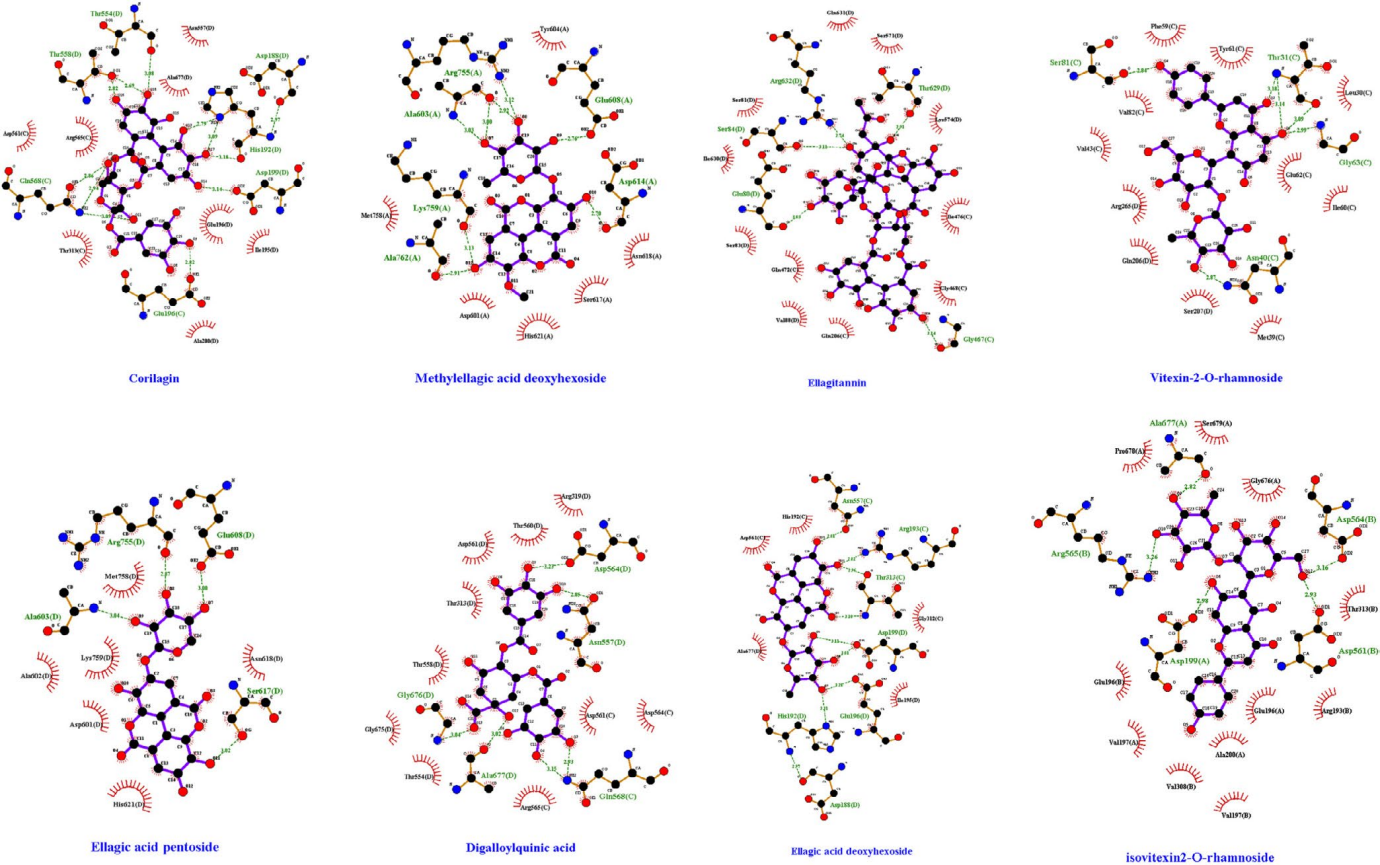
Static images of the interaction between the compound and the amino acid residues of PFK.

PFK is a key regulatory enzyme in glycolysis. Increased PFK activity promotes cancer cell proliferation (Lu et al. 2023). Our unpublished data indicate that PFK is the primary target of CO‐EtOAc, which binds to and inhibits the enzyme, thereby exerting its antiproliferative effects. To investigate whether small‐molecule compounds in CO water extracts interact with PFK, we combined the chemical profiles of Leaf‐WE, Fruit‐WE, and Pit‐WE for molecular docking analysis. ATP, a natural substrate of PFK, was included as a reference ligand for comparative binding affinity. As shown in Table 1, eight compounds exhibited more negative binding energies (kcal/mol) than did ATP, indicating stronger predicted binding. These compounds, listed in descending order of affinity, included corilagin, methylellagic acid deoxyhexoside, ellagitannin, vitexin 2‐*O*‐rhamnoside, ellagic acid pentoside, digalloylquinic acid, ellagic acid deoxyhexoside, and isovitexin 2‐*O*‐rhamnoside. Each compound also interacted with specific amino acid residues of PFK (Figure 7). By contrast, five additional compounds, such as methylellagic acid pentoside, dihydroxybenzoic acid apiosyl‐xyloside, theogallin, glucosyringic acid, and GA, exhibited weaker binding, with less negative binding energies. These findings indicated that certain compounds in CO water extracts directly interacted with PFK, contributing to their inhibitory effects on cancer cell proliferation.

**TABLE 1:** Binding energy and interacting residues of phenolic flavonoid compounds found in Chinese olive (CO) leaf, fruit, and pit water extracts against PFK.

Extracts	Compounds/ligands	Binding energy (Kcal/mol)	Interacting residues
Fruit‐WE	Corilagin	− 11.366	Asp188, His192, Glu196, Asp199, Thr554, Thr558, Gln568
Leaf‐WE Pit‐WE	Methylellagic acid deoxyhexoside	− 9.674	Ala603, Glu608, Asp614 Arg755, Lys759, Ala762
Fruit‐WE	Ellagitannin	− 9.351	Glu80, Ser84, Gly467, Thr629, Arg632
Leaf‐WE	Vitexin 2‐*O*‐rhamnoside	− 9.170	Thr31, Asn40, Gly63, Ser81
Leaf‐WE Fruit‐WE	Ellagic acid pentoside	− 9.166	Ala603, Glu608, Ser617, Arg755
Fruit‐WE	Digalloylquinic acid	− 9.147	Asn557, Asp564, Gln568, Gly676, Ala677
Fruit‐WE	Ellagic acid deoxyhexoside	− 9.139	Asp188, His192, Arg193, Glu196, Asp199, Thr313, Asn557
Leaf‐WE	Isovitexin 2‐*O*‐rhamnoside	− 8.823	Asp199, Asp561, Asp564, Arg565, Ala677
—	Adenosine triphosphate (ATP)^a^	− 8.525	Ligand for PFK1

^a^ ATP was used as a reference ligand.

### 3.1 Effects of CO Water Extracts on PFK Enzyme Activity in HCT116 Cells

**FIGURE 8:**
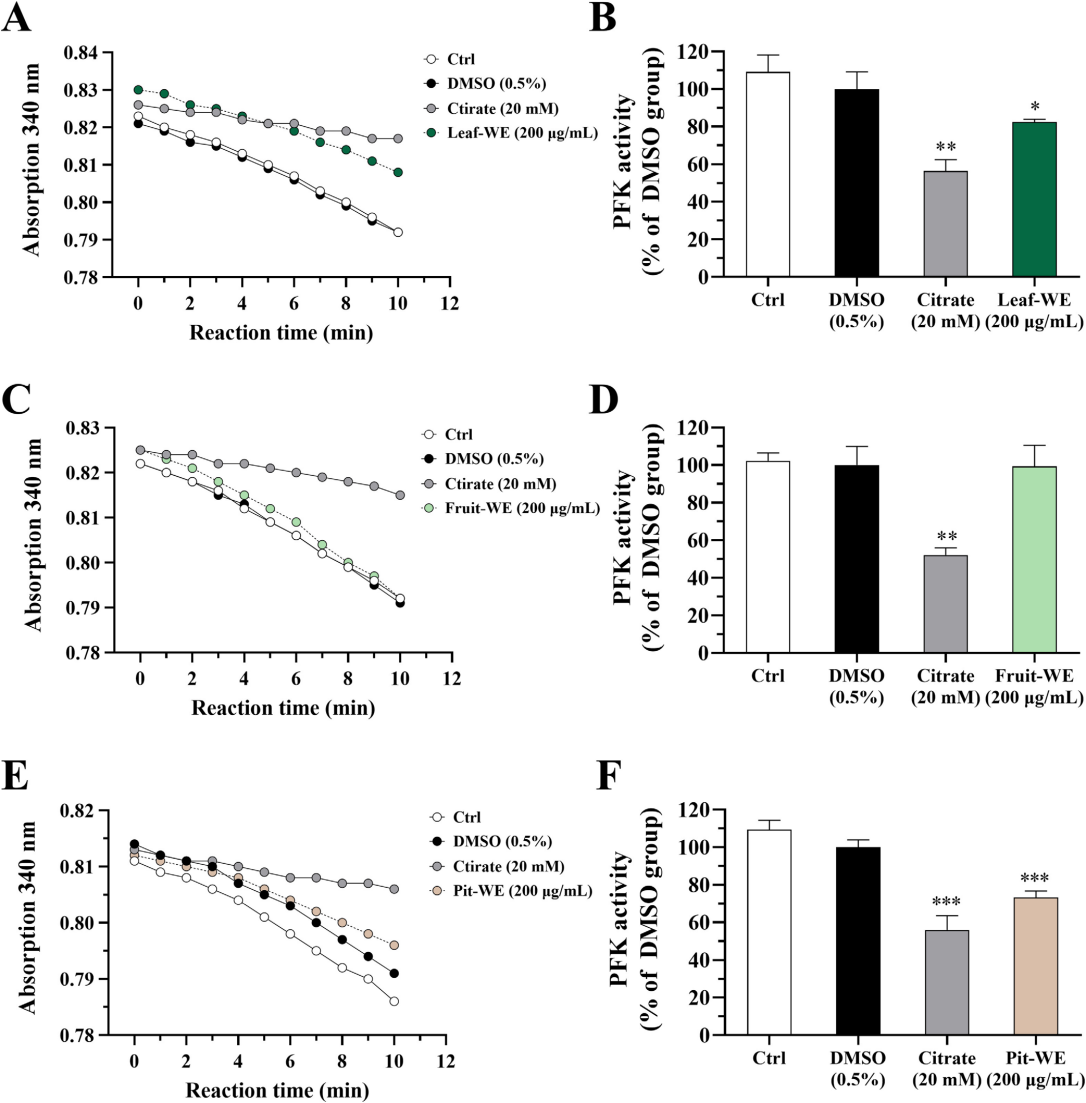
Effects of CO leaf, fruit, and pit water extracts on PFK activity in HCT116 cells. Cells were incubated with DMSO (0.5%; solvent control), sodium citrate (20 mM; as a positive control), or various CO water extracts (200 μg/mL) for 24 h. PFK activity was measured in cell lysates and normalized to total protein content. (A) Absorbance changes reflecting the conversion from NADH to NAD^+^ and (B) PFK activity in cells incubated with Leaf‐WE, (C, D) Fruit‐WE, or (E, F) Pit‐WE. Data are presented as mean ± SD values obtained from three independent experiments. (*) *p* < 0.05, (**) *p* < 0.01, and (***) *p* < 0.001 versus the DMSO group; unpaired two‐tailed Student's *t*‐test. CO, Chinese olive; WE, water extract.

**FIGURE 9:**
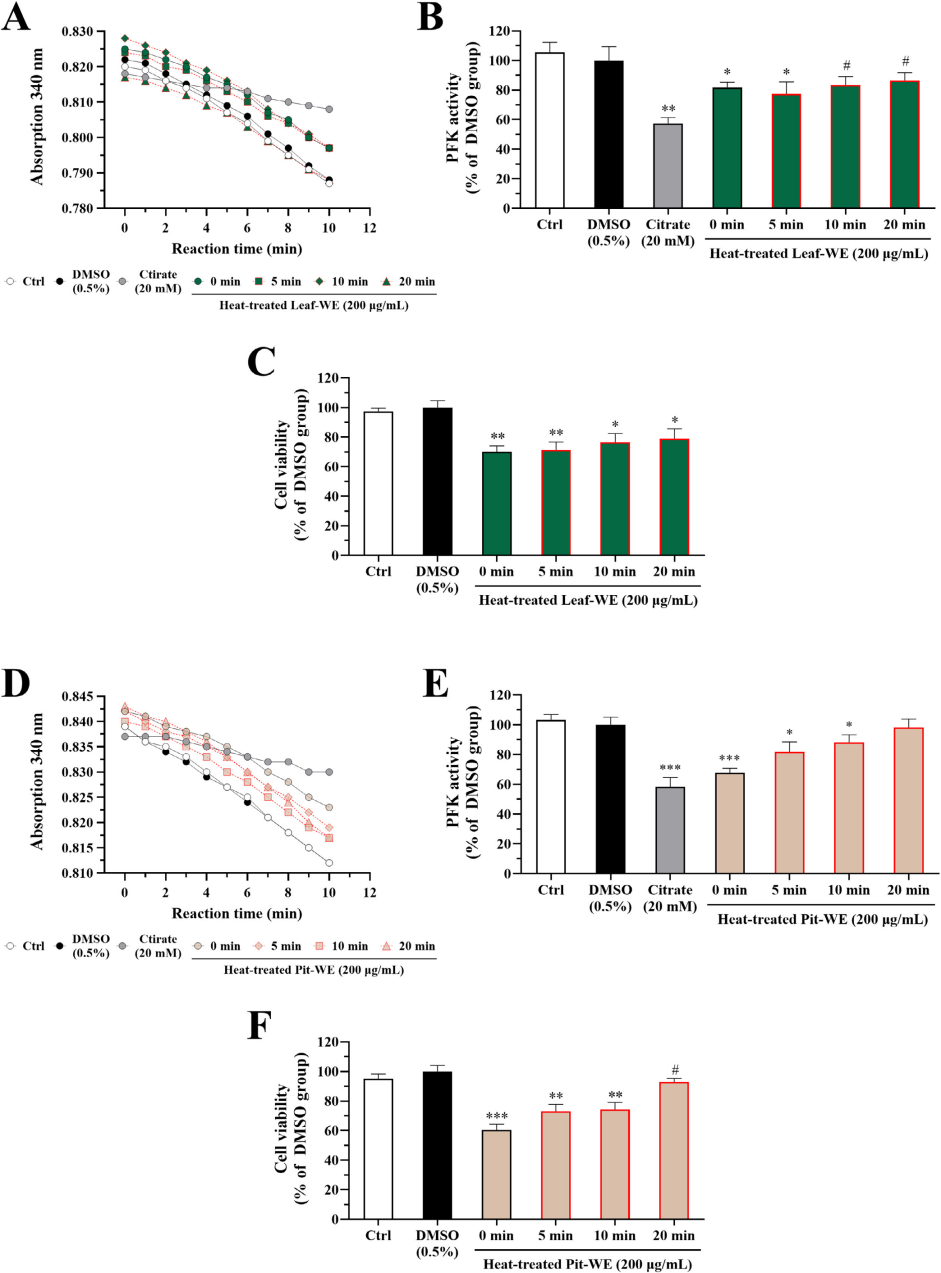
Effects of heat‐treated CO leaf and pit water extracts on PFK activity in HCT116 cells. Cells were incubated with DMSO (0.5%; solvent control), sodium citrate (20 mM; as a positive control), or various heat‐treated water extracts (200 μg/mL) for 24 or 72 h. PFK activity was measured in cell lysates and normalized to total protein content. (A) Absorbance changes reflecting the conversion from NADH to NAD^+^, (B) PFK activity, and (C) cell viability in cells incubated with Leaf‐WE or (D–F) Pit‐WE. Data are presented as mean ± SD values obtained from three independent experiments. (#) 0.05 < *p* < 0.1, (*) *p* < 0.05, (**) *p* < 0.01, and (***) *p* < 0.001 versus the DMSO group; unpaired two‐tailed Student's *t*‐test. Heating conditions: 100°C water bath for 0, 5, 10, or 20 min. CO, Chinese olive; WE, water extract.

We investigated whether CO water extracts affect PFK enzyme activity in colorectal cancer cells. HCT116 cells were treated with Leaf‐WE, Fruit‐WE, or Pit‐WE at a concentration of 200 μg/mL for 24 h. PFK activity was measured using a kinetic enzyme assay and normalized to total cellular protein content, as described in Section 2.10. Treatment with 0.5% DMSO (solvent control) did not alter PFK activity, whereas sodium citrate (20 mM), a known PFK inhibitor (Xia et al. 2018), significantly reduced enzyme activity by approximately 50%. Compared with the solvent control, PFK activity significantly decreased to approximately 70% in the Leaf‐WE group (Figure 8A,B), remained unchanged in the Fruit‐WE group (Figure 8C,D), and decreased to approximately 80% in the Pit‐WE group (Figure 8E,F). Furthermore, short‐term heating of Leaf‐WE (Figure 9A–C) and Pit‐WE (Figure 9D–F) did not reduce their inhibitory effects because both extracts continued to significantly reduce PFK activity and viability of HCT116 cells. These results suggested that CO water extracts, particularly those derived from the leaf and pit, suppressed HCT116 cell proliferation by inhibiting PFK activity.

## 4 Discussion

Our findings demonstrated the physiological effects and functional components of various edible parts of CO, supporting their potential for broader dietary applications. In this study, we prepared cooking‐mimicking extracts from CO leaves, fruits, and pits using water (WE), water/ethanol (1:1, v/v; WEE), ethanol (EE), and hexane (HE) as extraction solvents and also performed extract combinations and heat treatment to simulate conventional culinary practices and dietary conditions. The correspondence between extraction methods, simulated culinary conditions, and CO‐based dishes is summarized in Table S2. Concentration ranges for the CO extracts were determined through preliminary cytotoxicity assessments to ensure no adverse effects on the viability of RAW264.7 (Figure S1) and Ca9‐22 (Figure S2) cells. These predetermined concentrations were subsequently applied to evaluate anti‐inflammatory and antioxidant activities. The selected concentrations were adopted from dosages reported in our previous studies on CO‐EtOAc extract (Hsieh et al. 2016; Kuo et al. 2019).

NO is a crucial signaling molecule and a key mediator of inflammation. Inhibiting NO production in LPS‐stimulated RAW264.7 macrophages is widely accepted as a reliable platform for screening potential therapeutic agents targeting chronic inflammatory diseases. Our findings revealed that CO fruit extracts significantly suppressed NO production in RAW264.7 cells, consistent with the findings of studies demonstrating the anti‐inflammatory properties of phenolic compounds in CO‐EtOAc and Chinese CO cultivars (Kuo et al. 2019; He et al. 2024). To the best of our knowledge, this study is the first to demonstrate that extracts from CO leaves and pits significantly reduced NO production, exhibiting strong inhibitory effects even at low treatment concentrations. These results suggest that CO extracts exhibit potent anti‐inflammatory activity. Li, Wang, Wang, et al. (2022) reported that ethanol extracts of CO fruit inhibited NO production by downregulating the classical proinflammatory nuclear factor‐κB signaling pathway in macrophages. We previously demonstrated that CO‐EtOAc suppressed nuclear factor‐κB activity and its downstream target, cyclooxygenase‐2; reduced the expression and secretion of proinflammatory cytokines; and induced an anti‐inflammatory phenotype in macrophages (Yeh, Hsu, et al. 2023). These findings offer crucial insights for further investigation into mechanisms underlying the anti‐inflammatory properties of CO extracts. Given that chronic inflammation promotes tumor progression and plays a central role in cancer cell proliferation (Zhao et al. 2021), we speculate that the antiproliferative effects of CO extracts are partially attributable to their anti‐inflammatory capacity.

Free radicals, particularly reactive oxygen and nitrogen species, have been implicated in the pathogenesis of various human diseases (Chaudhary et al. 2023). Reactive oxygen and nitrogen species play a dual role in the host system, functioning as both harmful and beneficial agents depending on the context (Valko et al. 2007). Traditional assessments of antioxidant capacity are often conducted using in vitro chemical methods. For instance, Kuo et al. (2015) evaluated the antioxidant properties of CO fruit extracts by measuring their radical scavenging activity through the 2,2′‐azino‐bis(3‐ethylbenzthiazoline‐6‐sulfonic acid) and 1,1‐diphenyl‐2‐picryl‐hydrazyl assays. In the present study, we examined the transactivation activity of Nrf2, a key regulator of antioxidant responses, through a luciferase‐based reporter assay. Our findings indicated that none of the CO extracts enhanced Nrf2 transcriptional activity; most extracts exerted slightly inhibitory effects. Studies have reported that certain natural compounds activate the Nrf2 pathway, thereby reducing oxidative stress and inflammation and preventing colorectal cancer development (Li, Wang, Liu, et al. 2022). However, many studies indicated the adverse effects of Nrf2 activation in cancer—for example, the promotion of tumor growth and induction of resistance to chemotherapeutic agents (Wang et al. 2014; Huang et al. 2023). Therefore, the reduced Nrf2 activation observed following CO extract treatment may suggest its role in enhancing the effectiveness of chemotherapy, although further investigation is required to confirm this hypothesis.

Researchers have identified various polyphenolic compounds in the leaves of fruit trees such as apple ( *Malus domestica* Borkh.), blackcurrant ( *Ribes nigrum* L.), cranberry ( *Vaccinium macrocarpon* L.), and guava ( *Psidium guajava* L.) (Teleszko and Wojdyło 2015; Kumar et al. 2021). However, research on the physiological functions and bioactive components of CO leaves and pits remains limited. Xiao et al. identified canaroleoside A–C, myricetin, quercetin, kaempferol, and urolithin M5 in the ethyl acetate–butanol fractions of CO leaves and demonstrated their inhibitory effects on neuraminidase activity against influenza viruses (Xiao, Cao, et al. 2022; Xiao, Kong, et al. 2022). In the present study, we report for the first time that CO leaf extracts exert antiproliferative effects on HCT116 cells. LC–MS/MS analysis identified methylellagic acid deoxyhexoside, vitexin 2‐*O*‐rhamnoside, and isovitexin 2‐*O*‐rhamnoside in Leaf‐WE. Zhang et al. (2014) demonstrated that an ellagic acid derivative inhibited proliferation and induced G1/S phase cell cycle arrest in HepG2 hepatocellular carcinoma cells. Additionally, plant extracts containing vitexin 2‐*O*‐rhamnoside suppressed the synthesis of DNA in MCF‐7 breast cancer cells (Ninfali et al. 2017), induced death in Hep3B HCC cells (Ninfali et al. 2007), and inhibited the proliferation of HT29 colorectal adenocarcinoma and HeLa cervical cancer cells (Sowa et al. 2023). Isovitexin 2‐*O*‐rhamnoside exhibited anticancer activity against human stomach cancer cells (Ribeiro et al. 2023). These findings may support the view that CO leaf extracts possess antiproliferative properties against HCT116 cells and hold promise in dietotherapy.

Among all CO extracts tested, Leaf‐WEE and Leaf‐EE exhibited the most pronounced antiproliferative effects. This might be attributable to their elevated total PFC content because ethanol extracts contained more than four times the PFC levels found in Leaf‐WE. Specifically, methylellagic acid deoxyhexoside concentrations in Leaf‐WEE and Leaf‐EE were approximately two‐ and three‐fold higher, respectively, than in Leaf‐WE. In addition, Leaf‐EE contained ellagitannin‐3 and ellagitannin‐4, which were absent in Leaf‐WE. A similar pattern was observed in CO fruit extracts. Both Fruit‐WEE and Fruit‐EE significantly inhibited HCT116 cell proliferation and contained ellagitannin‐3 at levels approximately twice those in Fruit‐WE. Given the well‐documented anticancer activity of ellagitannins (Ross et al. 2007; Heber 2008; Kasimsetty et al. 2010; Sharma et al. 2010; Senobari et al. 2022), their relatively low abundance in Fruit‐WE may explain its limited antiproliferative effect. Previous investigations of CO‐EtOAc extracts identified GA, ellagic acid, and several novel phenolic compounds with antiproliferative properties (He and Xia 2007b; Yeh, Chen, et al. 2023). In our analysis, however, theogallin (3‐*O*‐galloylquinic acid) was identified as the major compound in Fruit‐WE, Fruit‐WEE, and Fruit‐EE, comprising > 80% of the total extract content. Theogallin has been previously identified in CO fruits, tea leaves, and *Arbutus unedo* L. fruits (Chang et al. 2017; Cartwright and Roberts 1954; Pawlowska et al. 2006). A recent study also reported that theogallin exhibits promising antineoplastic activity and enhances chemosensitivity to doxorubicin (Abd El‐Salam et al. 2022). These findings suggest that solvent polarity affects the composition of extracted components; as polarity decreases from WE to EE, theogallin content declines while ellagitannin levels increase. Our study provides a more comprehensive understanding of bioactive constituents in different edible parts of CO and highlights the potential dietary therapeutic value of WEE and EE extracts.

To the best of our knowledge, no studies have examined the chemical composition of CO pits thus far. Notably, our findings revealed that CO pit extracts significantly reduced the viability of HCT116 cells. Although the total PFC content in CO pits was lower than that in CO leaves and fruits, except for Pit‐WE, this may reflect their relatively higher lipid content. Nevertheless, Pit‐WE contained two key anticancer compounds: GA and methylellagic acid deoxyhexoside. GA exerts anticancer effects by inhibiting cell migration, metastasis, angiogenesis, and oncogene expression while inducing cell cycle arrest (Jiang et al. 2022). Additionally, GA inhibits colony formation and induces apoptosis in HCT‐15 colorectal cancer cells (Subramanian et al. 2016) and HCT116 cells through calcium ion channels and the p53 signaling pathway (Yang, Xei, et al. 2018). Notably, none of the 12 CO extracts examined in the present study exerted cytotoxic effects on human normal colon epithelial cells at the highest tested concentration (200 μg/mL). This finding highlights their potential safety and selectivity for use in dietary cancer therapy.

A recent open pilot study reported that a food supplement containing *Olea europaea* L. leaf and fruit extracts improved blood pressure and metabolic syndrome markers in individuals with prehypertension (Hermans et al. 2020). Given that CO leaves, fruits, and pits are conventionally used in Asian cuisine, especially by water‐based methods to prepare teas and soups such as olive tea and olive chicken soup (Kuo et al. 2015), we examined their combined anticancer effects. Our findings revealed that combinations of Leaf‐WE and Fruit‐WE (mimicking olive tea); Fruit‐WE and Pit‐WE (mimicking olive soup); and Leaf‐WE, Fruit‐WE, and Pit‐WE (also mimicking olive soup) significantly reduced HCT116 cell viability compared with the effects of individual treatments. These results suggest that CO WEs synergistically inhibit the proliferation of colorectal cancer cells.

PFK is a key regulatory enzyme in glycolysis, and its inhibition disrupts glucose metabolism and suppresses cancer cell proliferation (Xia et al. 2018). Molecular docking analysis revealed that the three major compounds in Leaf‐WE exhibited stronger binding affinity for PFK, as indicated by more negative binding energy levels than that with ATP. These findings suggest that the antiproliferative effects of Leaf‐WE are associated with PFK inhibition. Although Fruit‐WE contained several compounds capable of binding to PFK—for example, corilagin, ellagitannins, ellagic acid pentoside, digalloylquinic acid, and ellagic acid deoxyhexoside, Fruit‐WE did not significantly inhibit HCT116 cell proliferation. Corilagin, which exhibited the strongest binding affinity for PFK, suppresses the growth of colorectal, liver, lung, and breast cancer cells (Deng et al. 2018; Zheng et al. 2016; Bai et al. 2019; Tong et al. 2018). However, it was present only in trace amounts in Fruit‐WE. By contrast, theogallin, the most abundant compound in both Fruit‐WE and Pit‐WE, exhibited lower binding affinity for PFK than did ATP. Notably, Pit‐WE contained high levels of GA and methylellagic acid deoxyhexoside, both of which have been linked to a substantial reduction in cancer cell viability (Zhang et al. 2014). Furthermore, both Leaf‐WE and Pit‐WE, including their heat‐treated forms, significantly inhibited PFK activity and viability in HCT116 cells, supporting the hypothesis that functional components in CO act synergistically to suppress PFK activity and inhibit cancer cell proliferation.

Although CO leaves and pits are generally regarded as inedible and are rarely consumed on their own, they become suitable for consumption following certain culinary processes such as brewing, stewing, soaking in wine, or oil‐based pickling. In light of the observed anti‐inflammatory and anticancer properties of CO extracts and their combinations, we propose that the edible parts of CO should be widely integrated into daily diets as natural health‐promoting foods. To maximize their functional benefits, we recommend the following practices: (1) combine CO leaves, fruits, and pits in varied proportions; (2) cut or grind the fruits and pits prior to cooking; (3) soak the raw materials in water for an extended period before cooking; (4) limit heating time to 5–10 min to preserve thermolabile active components; and (5) add a small amount of alcohol to enhance the extraction of bioactive compounds.

## 5 Conclusion

**FIGURE 10:**
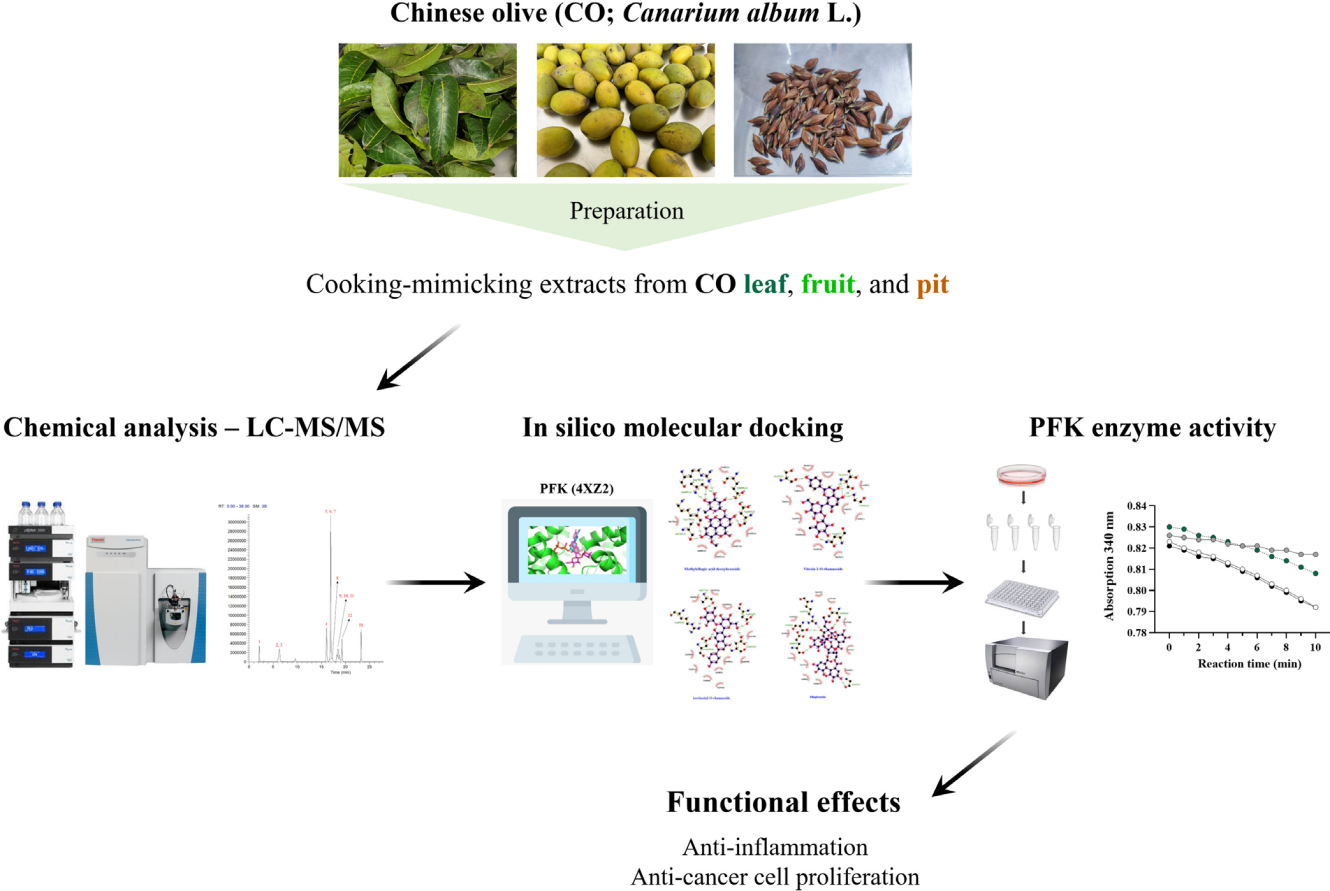
Bioactive components from Chinese olive extracts demonstrate anti‐inflammatory properties and inhibit human colorectal cancer cell proliferation by suppressing phosphofructokinase enzyme activity.

We prepared extracts from CO leaves, fruits, and pits under conditions simulating conventional culinary practices and real dietary consumption and evaluated their functional properties. Most CO extracts exhibited anti‐inflammatory and antiproliferative effects against cancer cells, although they did not activate the Nrf2‐mediated antioxidative pathway. Notably, combinations of CO WEs exhibited synergistic effects in suppressing colorectal cancer cell proliferation. In addition, we identified key bioactive compounds in the CO extracts and proposed a potential mechanism of action involving the inhibition of PFK activity (Figure 10). These findings provide valuable insights into the functional components and physiological effects of CO leaves, fruits, and pits, contributing to a broader understanding of their potential in dietotherapy.

## Author Contributions

**Chun‐Wai Chan:** conceptualization (equal), formal analysis (equal), investigation (equal), methodology (equal), software (equal), visualization (equal), writing – original draft (lead), writing – review and editing (equal). **Yu‐Jo Tsai:** conceptualization (equal), formal analysis (equal), investigation (equal), methodology (equal), software (equal), visualization (equal), writing – original draft (supporting), writing – review and editing (equal). **Ting‐Jang Lu:** methodology (supporting), resources (supporting), writing – review and editing (supporting). **Yi‐Chun Liao:** data curation (supporting), resources (supporting), validation (supporting), writing – review and editing (supporting). **Shu‐Chen Hsieh:** conceptualization (equal), data curation (lead), validation (lead), supervision (lead), writing – original draft (lead), writing – review and editing (equal), project administration (lead), funding acquisition (lead). All authors have read and approved the manuscript.

## Conflicts of Interest

The authors declare no conflicts of interest.

## Supporting information


Data S1.

## Data Availability

Data available on request from the authors.
