# Correction to “Functional Evaluation of Cooking‐Mimicking Extracts From Chinese Olive (*Canarium album* L.) Leaves, Fruits, and Pits Using Cell‐Based and In Silico Analysis”

**DOI:** 10.1002/fsn3.70492

**Published:** 2025-06-23

Chan, C. W., Tsai, Y. J., Lu, T. J., Liao, Y. C., & Hsieh, S. C. (2025), Functional Evaluation of Cooking‐Mimicking Extracts From Chinese Olive (*Canarium album* L.) Leaves, Fruits, and Pits Using Cell‐Based and In Silico Analysis. *Food Sci. Nutr*., 13(6), e70337. https://doi.org/10.1002/fsn3.70337

In Figure 1, the abbreviations of the CO extracts are incorrect. Here is the corrected figure.

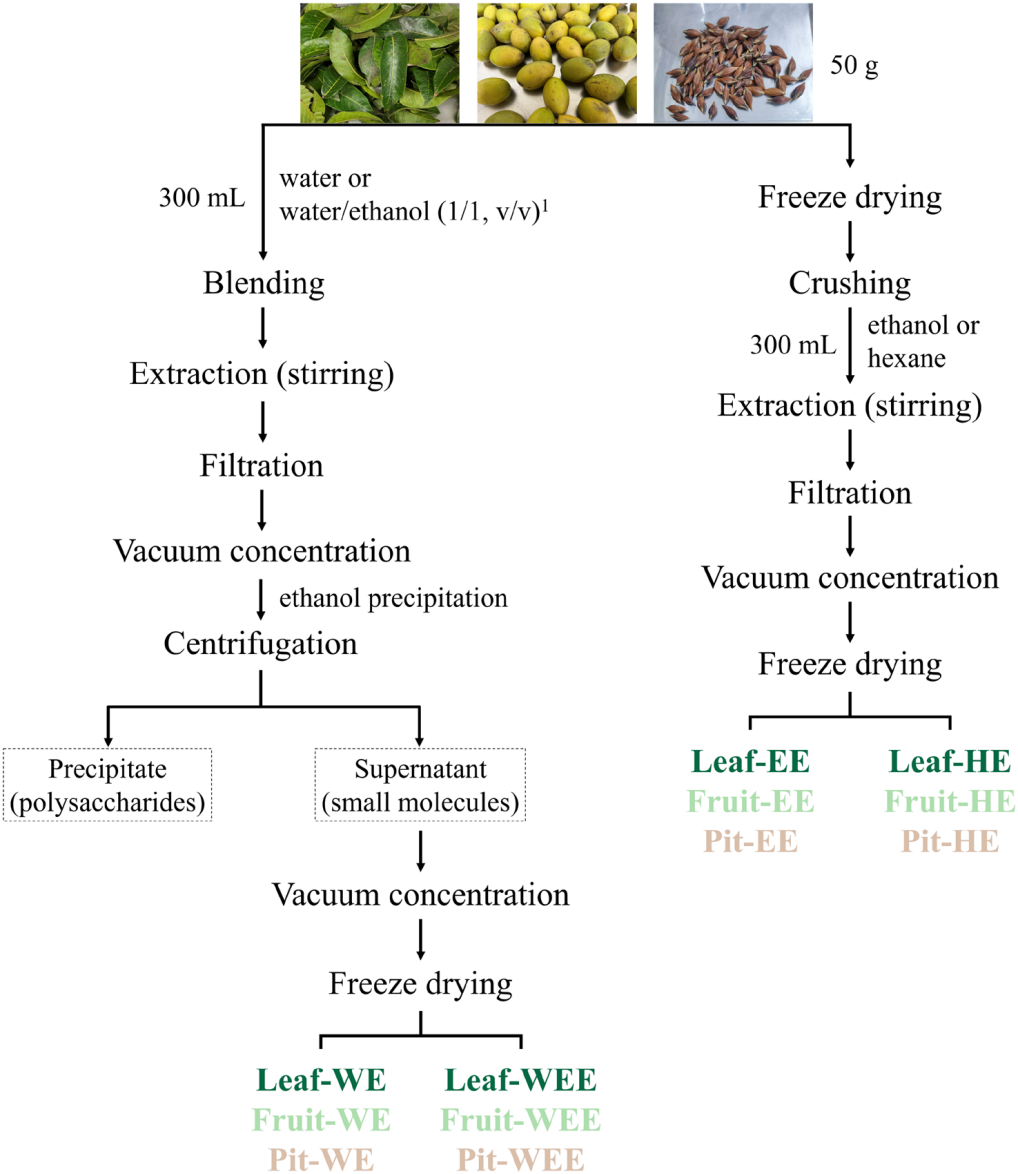

We apologize for this error.

