# Supplementary material for: Functional Evaluation of Cooking‐Mimicking Extracts From Chinese Olive ( Canarium album L.) Leaves, Fruits, and Pits Using Cell‐Based and In Silico Analysis
Source: Food Sci Nutr. 2025 May 28;13(6):e70337. doi: 10.1002/fsn3.70337 (PMC12121516; doi:10.1002/fsn3.70337)
Supplement: Supplementary file 1 — Data S1. [file FSN3-13-e70337-s001.docx]

**Functional Evaluation of Cooking-Mimicking Extracts From Chinese Olive (*Canarium album* L.) Leaves, Fruits, and Pits Using Cell-Based and In Silico Analysis**

Chun-Wai Chan^1^, Yu-Jo Tsai^1^, Ting-Jang Lu^1^, Yi-Chun Liao^2^, and Shu-Chen Hsieh*^1^

Chun-Wai Chan and Yu-Jo Tsai contributed equally to this work.

^1^*Institute of Food Science and Technology, College of* *Bioresources and Agriculture,* *National Taiwan University,* *No. 1, Sec. 4, Roosevelt Rd., Taipei 106, Taiwan*

^2^*Department of Biochemical Science and Technology, College of Life Science, National Taiwan University, Taipei 106, Taiwan*

*Corresponding author

**Shu-Chen Hsieh**

*Institute of Food Science and Technology, College of Bioresources and Agriculture, National Taiwan University, No. 1, Sec. 4, Roosevelt Rd., Taipei 106, Taiwan*;

Phone: 886-2-3366-9871; Fax: 886-2-2362-0849; E-mail: [scjhsieh@ntu.edu.tw](mailto:scjhsieh@ntu.edu.tw)

**Supporting Information**

**Table S1.** Percentage yield of Chinese olive (CO) leaf, fruit, and pit extracts.

| **CO extracts** | **Solvent** | **Dry Weight (g)** | **Yield (%)** ^a^ |
| --- | --- | --- | --- |
| Leaf-WE | Water | 0.97 | 1.94 |
| Leaf-WEE | Water/Ethanol (1/1, v/v) | 1.67 | 3.34 |
| Leaf-EE | Ethanol | 0.84 | 1.68 |
| Leaf-HE | Hexane | 0.19 | 0.38 |
| Fruit-WE | Water | 1.84 | 3.68 |
| Fruit-WEE | Water/Ethanol (1/1, v/v) | 0.43 | 0.86 |
| Fruit-EE | Ethanol | 0.93 | 1.86 |
| Fruit-HE | Hexane | 0.61 | 1.22 |
| Pit-WE | Water | 0.18 | 0.36 |
| Pit-WEE | Water/Ethanol (1/1, v/v) | 0.32 | 0.64 |
| Pit-EE | Ethanol | 0.37 | 0.74 |
| Pit-HE | Hexane | 2.30 | 4.60 |

^a^ Yield (%) represents the ratio of the weight (g) of each extract to 50 g of fresh CO leaf, fruit, and pit.

EE, ethanol extract; HE, hexane extract; WE, water extract; WEE, water/ethanol (1/1, v/v) extract.

**Table S2.** Correspondence between extraction methods, simulated culinary conditions, and CO-based dishes

| **Extraction Methods** | **Simulated**  **Culinary Conditions** | **Edible Part**  **of CO** | **Simulated**  **CO-Based Dishes** |
| --- | --- | --- | --- |
| **WE** | brewing tea  boiling soup | leaf, fruit, pit  leaf, fruit, pit | “CO tea”  “CO soup” |
| **WEE** | boiling soup | leaf, fruit, pit | “CO chicken soup” |
| **EE** | soaking wine  boiling soup | fruit, pit  leaf, fruit, pit | “CO wine”  “CO chicken wine soup” |
| **HE** | oil-pickle soaking  boiling soup | fruit, pit  leaf, fruit, pit | “oil-soaking CO”  “CO chicken soup” |

CO, Chinese olive; EE, ethanol extract; HE, hexane extract; WE, water extract; WEE, water/ethanol (1/1, v/v) extract.

**Figure S1.** Effects of CO leaf, fruit, and pit extracts on cell viability of RAW264.7 macrophages. Cells were incubated with dimethyl sulfoxide (DMSO, 0.5%; solvent control) or various concentrations (25, 50, 100, and 200 μg/mL) of CO (A) leaf, (B) fruit, or (C) pit extracts and cotreated with lipopolysaccharide (LPS, 100 ng/mL) for 24 h. Cell viability was assessed using the MTT assay. Data are presented as mean ± standard deviation (SD) values obtained from four independent experiments. CO, Chinese olive; EE, ethanol extract; HE, hexane extract; WE, water extract; WEE, water/ethanol (1/1, v/v) extract.

**Figure S2.** Effects of CO leaf, fruit, and pit extracts on cell viability of ARE−luciferase Ca9-22 cells. Cells were incubated either with DMSO (0.5%; solvent control), *tert*-butylhydroquinone (*t*-BHQ, 10 μM; positive control); or various concentrations (50, 100, and 200 μg/mL) of CO (A) leaf, (B) fruit, or (C) pit extracts for 24 h. Cell viability was assessed using the MTT assay. Data are presented as mean ± SD values obtained from four independent experiments. CO, Chinese olive; EE, ethanol extract; HE, hexane extract; WE, water extract; WEE, water/ethanol (1/1, v/v) extract.

**Figure S3.** Effects of CO leaf, fruit, and pit extracts on cell viability of CCD841 CoN human normal colon epithelial cells. Cells were incubated either with DMSO (0.5%; solvent control) or various CO extracts (200 μg/mL) for 72 h. Cell viability was assessed using the MTT assay. Data are presented as mean ± SD values obtained from three independent experiments. CO, Chinese olive; EE, ethanol extract; HE, hexane extract; WE, water extract; WEE, water/ethanol (1/1, v/v) extract.
